# Detection and identification of drug traces in latent fingermarks using Raman spectroscopy

**DOI:** 10.1038/s41598-022-07168-6

**Published:** 2022-02-24

**Authors:** Mohamed O. Amin, Entesar Al-Hetlani, Igor K. Lednev

**Affiliations:** 1 Department of Chemistry, Kuwait University, Faculty of Science, P.O. Box 5969, 13060 Safat, Kuwait; 2 Department of Chemistry, University at Albany, SUNY, 1400 Washington Avenue, Albany, NY 12222 USA

**Keywords:** Biochemistry, Bioanalytical chemistry, Microscopy, Raman spectroscopy

## Abstract

Recent advancements in analytical techniques have greatly contributed to the analysis of latent fingermarks’ (LFMs) “touch chemistry” and identification of materials that a suspect might have come into contact with. This type of information about the FM donor is valuable for criminal investigations because it narrows the pool of suspects. It is estimated that at least 30 million people around the world take over-the-counter and prescription nonsteroidal anti-inflammatory drugs (NSAIDs) for pain relief, headaches and arthritis every day. The daily use of such drugs can lead to an increased risk of their abuse. In the present study, Raman spectroscopy combined with multivariate statistical analysis was used for the detection and identification of drug traces in LFMs when NSAID tablets of aspirin, ibuprofen, diclofenac, ketoprofen and naproxen have been touched. Partial least squares discriminant analysis of Raman spectra showed an excellent separation between natural FMs and all NSAID-contaminated FMs. The developed classification model was externally validated using FMs deposited by a new donor and showed 100% accuracy on a FM level. This proof-of-concept study demonstrated the great potential of Raman spectroscopy in the chemical analysis of LFMs and the detection and identification of drug traces in particular.

## Introduction

Fingerprints are among the greatest discoveries of forensic sciences and have been employed for decades as the primary biometric means of human individualization. Establishment of a clear connection between a questioned fingermark (FM) and an individual entails a minute comparison of the morphological pattern of the FM with a reference material (e.g., a fingerprint from a suspect)^1^. However, this forensic application of FM is based on the comparison with a known FM either collected from a suspect or found in the database. The biochemical composition of a FM contains numerous information about the donor, including age^2^, sex^3^, health status^4^ and other characteristics that may have been previously overlooked. Such characteristics can be identified by the presence of thousands of compounds in FM residue originating from both endogenous and exogenous components. Endogenous substances result from the natural secretion of the human body, such as lipids, waxes, amino acids, and proteins, while substances that come in contact with fingertips prior to deposition (e.g., drugs, cosmetics, explosives) are often referred to as exogenous substances. In addition, semi-exogenous substances can also be found in FMs; these substances result from compounds that are ingested and then excreted in their metabolized and unmetabolized forms through sweat (e.g. drugs, food and drink components)^5^.

These exogenous substances reflect information about the individual’s lifestyle and therefore have been the focus of several recent studies^6^. Thus, chemical analysis of the contaminants in FMs has served as a mainstay in forensic investigations, producing prosecutorial evidentiary data^5^. For this purpose, several analytical techniques have been employed for the chemical analysis of FMs, such as gas chromatography-mass spectrometry (GC–MS)^7^, ultra-performance liquid chromatography-mass spectrometry (UPLC–MS)^8^, laser desorption/ionization-mass spectrometry (LDI-MS)^9^ and others.

Owing to their inherent nondestructive nature and the need for little to no sample pretreatment, vibrational spectroscopic techniques, including Raman spectroscopy, Fourier transform infrared (FTIR) spectroscopy and attenuated total reflection Fourier transform infrared (ATR-FTIR) spectroscopy, have great potential in forensic applications^10–12^. Vibrational spectroscopy has garnered a considerable amount of attention in the field of FM analysis^13^. A comprehensive review by Ewing and Kazarian discussed the use of vibrational spectroscopic imaging to obtain the chemical composition of FMs and exogenous materials^14^. In particular, ATR-FT-IR spectroscopic imaging has been utilized for the analysis of latent FM and its changes under controlled humidity and temperature^15,16^. Furthermore, Kazarian and coworkers have investigated the use of common adhesive tape and polydimethylsiloxane (PDMS) film along with ATR spectroscopy to detect drugs of abuse^17^. Commercial gel lifters and ATR-FTIR spectroscopy have been used to generate chemical imaging from a different depth of FMs^18^. In addition, FM contaminated with cosmetics on porous and nonporous surfaces have been analyzed^19^.

Raman spectroscopy, through its technological advancement, has enabled efficient detection of several forensically relevant contaminants and household products in FM. For instance, significant strides have been made in the detection of explosive residues in FMs, which is of importance in preventing terroristic attacks on civilians^20,21^. In addition, drug-contaminated FMs are valuable evidence that can be used to reconstruct various types of crime scenes, such as suicide, drug abuse, drug manufacturing and others. In this regard, Raman spectroscopy has been applied in two different studies that detected drugs of abuse^22^, including codeine, cocaine, amphetamine, barbital and nitrazepam and adulterants, as well as caffeine, aspirin, paracetamol, starch and talc powders^23^ in FM impressions. Furthermore, FMs contaminated with the drugs of abuse including ecstasy, cocaine, ketamine and amphetamine have been lifted with adhesive tape and analyzed using Raman spectroscopy^24^. In another study by West and coworkers^25^, Raman spectroscopy was employed for the analysis of FMs contaminated with over-the-counter (OTC) analgesic substances. These FMs had been treated with powder and lifted with adhesive tape. The findings indicated that the application of powders did not hinder the identification of contaminants in FM impressions. Additionally, analysis of drug powder and additive doping in FMs was investigated using tape lifting and Raman microscopy; the obtained spectra were deconvoluted using the multivariate technique Band-target entropy minimization (BTEM)^26^. These combined methods enabled the identification of the test substances using their characteristic Raman signatures. Based on the above literature, limited studies have focused on the analysis of drug-contaminated FMs using Raman spectroscopy despite its importance in forensic cases.

This study expands the potential use of Raman spectroscopy for detecting drugs in contaminated FMs obtained by gentle touching nonsteroidal anti-inflammatory drug (NSAIDs) pharmaceutical tablets purchased from a local drugstore. Two important aspects of this approach for preparing mock contaminated FMs are considered. (1) NSAIDs pharmaceutical tablets contain active ingredient(s) as well as other excipients that makes the mock samples more realistic. (2) Touching a tablet is practically important and further expands the variety of drug-contaminated FMs, which can be analyzed by Raman spectroscopy. Five commonly used NSAID tablets were selected for this study and subjected to Raman analysis. These drugs share many of the same functional groups, and subtle differences can be seen in their Raman spectra when they are present in FMs. Therefore, the differentiation of FM samples was evaluated using multivariate data analysis. Initially, principal component analysis (PCA) was carried out on the preprocessed spectra for the removal of multivariate outliers, defined as spectra with high Hoteling’s T^2^ and Q residuals values in all the datasets, and they were removed before any further statistical analysis was performed. Further advanced statistical analysis was applied, including a genetic algorithm (GA), to select the most informative features in the spectra in the training process of partial least squares-discriminate analysis (PLS-DA) to distinguish between FM classes. Both internal and external validations were applied to evaluate the performance of the model. The obtained results clearly demonstrate that Raman spectroscopy is a powerful analytical method that can detect traces of drugs in FMs and define the type of drug with which the FM made contact.

## Experimental

### Sample preparation

Initially, hands were washed with water and soap and then thoroughly dried. The donor was requested to rub his index finger on the forehead, nose and chin five times to eventually produce a sebum-rich mark and to stimulate natural grooming behavior, after which the finger was placed directly on a microscope slide covered with aluminum adhesive tape (Nashua tape, Home Depot). The NSAID-contaminated FMs were prepared as follows: the donor was requested to rub his index on the forehead, nose and chin five times and then touch a pharmaceutical tablet purchased from a local drugstore containing one of these active ingredients: aspirin (500 mg), ibuprofen (400 mg), diclofenac (50 mg), ketoprofen (100 mg) or naproxen (500 mg) for 10 s. The contaminated FMs were applied to a microscope slide covered with aluminum tape for Raman spectroscopic analysis. All FMs were analyzed within 30 min post collection. All procedures were approved by the Health Sciences Centre Ethical Committee of Kuwait University and in accordance with the ethical standards of the institutional and/or national research committee and with the 1964 Helsinki Declaration and its later amendments or comparable ethical standards. An informed consent was obtained from all subjects and/or their legal guardian(s).

### Instrumentation and spectra collection

Spectra of the FMs were acquired utilizing a Renishaw inVia confocal Raman microscope with a 1200 L/mm grating and a CCD camera. The analyses were carried out using a 785 nm diode laser for excitation, and the spectra were collected in the range of 400 to 1800 cm^−1^, employing 50% laser power and 20 s exposure time. A longitudinal spot was attained at a high optical magnification of xL50, and WiRe 4.4 software was used to control the microscope. The instrument was calibrated with a silicon standard prior to analysis, and a vibrational band corresponding to a silicon wafer was used for this purpose. An automatic mapping stage was used for the analysis, and a total of 52–56 spectra were obtained from different spots to account for sample heterogeneity.

### Data analysis

Dataset preparation and statistical analysis were carried out using PLS Toolbox 8.9.1 (Eigenvector Research, Inc., Wenatchee, WA) operating in MATLAB R2020b (MathWorks, Inc., Natick, MA)^27^. All the spectra were baseline-corrected by automatic weighted squares, normalized and mean centered. A PCA model was constructed to identify multivariate outliers, which were removed before any further statistical analyses. Genetic algorithm (GA) was used to determine the main spectral features to be included in the training process of PLS-DA. A population size of 64 and generation number of 100 were used in the GA spectral selection. Double crossover was set for the breeding crossover rule, and the mutation rate was 0.005. A supervised learning technique, PLS-DA, was applied to distinguish between different FM classes and identify the drug(s) present in the questioned FMs.

### Validation tests

In this study, the internal validation of the PLS-DA model was carried out using the Venetian blinds method of cross validation (CV) with ten splits. In theory, the internal CV allows for testing how well the available dataset supports the classification hypothesis. A true validation of a classification model requires its testing on an external data, which is not used for building the model and has a size of at least 30–40% of the training dataset. For these reason, we conducted both an internal CV and external validation. The Venetian blinds CV involved a sequence of steps, in which a subset of ten spectra was removed from the training dataset and a sub-model was constructed using the remaining spectra. As a result, each sub-model was tested with spectra, which have not been used to build the model. The iterations were continued until each spectrum in the training dataset was removed once.

To further increase the reliability of the proposed method, an external validation using an independent dataset was employed. In this study, natural and contaminated FMs were obtained from a second donor following the same procedure mentioned above. The spectra were baseline-corrected by automatic weighted squares, normalized and mean centered and introduced to the PLS-DA model for prediction purposes. This validation demonstrated that the classification model built based on contaminated FMs obtained from one donor can be reliably applied for determining the NSAID drugs in contaminated FMs obtained from another donor.

## Results and discussion

### Spectral analysis of NSAIDs contaminated FM

FM residue is a complex mixture composed of numerous compounds originating from different sources and includes organic constituents (e.g., protein, lipids, vitamins and amino acids), ions (Cl^−^, K^+^, Na^+^, Mg^2+^) and trace metals (Zn, Cu, Fe) resulting from the natural secretions of the skin^5^. While several factors can influence the chemical composition of FMs such as donor, environmental conditions, type of substrate, and substances that may have come in contact with the fingertips, such as drugs, cosmetics and explosives. Specifically, drug-contaminated FMs can be discovered in a variety of crime scenes, including suicide cases, drug abuse, drug handling or street drug diluents that pertain to several different forensic scenarios. Due to the large number of drugs that can be employed for these purposes, we selected NSAIDs tablets for our proof-of-concept study because they are common drugs that can be found in any household and can be purchased OTC without a prescription at a low cost. Additionally, despite NSAIDs are supposed to be safe drugs, they may lead to severe toxic effects in cases of acute overdosage and chronic abuse. NSAIDs misuse has been also reported in horse doping, therefore, they may be encountered in clinical, forensic toxicological analyses and in horse doping control^28^.

Aluminum foil with an adhesive layer has been used to cover a glass slide and deposit the FMs Aluminum foil is an ideal substrate for Raman spectroscopy because it is readily available, inexpensive and most importantly it does not result in any significant interfering signal^29,30^. A minimal (gentle) pressure was applied on the tablet by the fingers, which was just sufficient to hold the tablet, to mimic a real-life situation. A holding time of about 10 s was used to make mock FMs samples. Although we did not investigate these two factors (pressure and time) quantitatively, no inconsistency or irreproducibility were noticed when the Raman spectra were analyzed. Most importantly, we were able to develop accurate classification model for drug differentiation.

**Figure 1:**
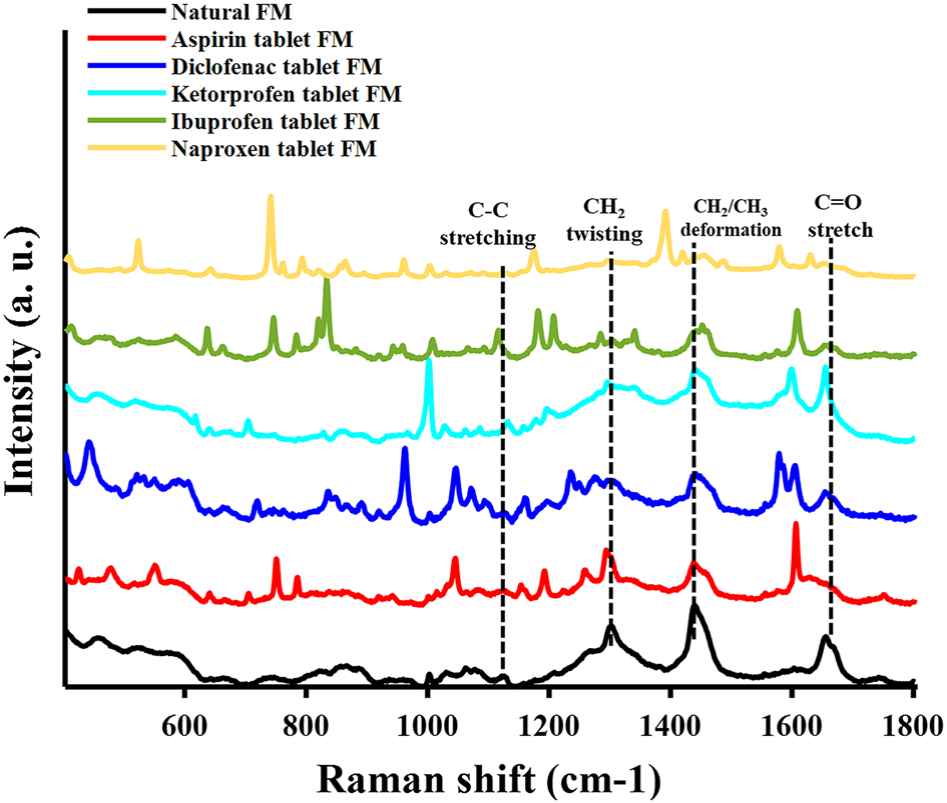
The average Raman spectra of natural fingermark and fingermarks contaminated with aspirin, diclofenac, ketoprofen, ibuprofen or naproxen tablets. The experimental spectra were preprocessed by baseline correction and normalization.

Initially, FMs contaminated with aspirin were obtained from all five fingers of the left hand of one donor. Average Raman spectra collected from each of those FMs (Figure S1) are similar to each other indicating that the intra-donor variability has minimum effect. Consequently, the analysis of FMs contaminated with various drugs was conducted in this study using the index finger only. In this study, Raman spectroscopy was employed to detect traces of NSAID tablets in FMs and distinguish between natural FM components and FMs after contact with different NSAID tablets. Five pharmaceutical tablets containing aspirin, ibuprofen, diclofenac, ketoprofen and naproxen as active ingredients were utilized to produce NSAID-contaminated FMs. 54–56 Raman spectra were obtained from each FM sample using automatic mapping to account for the inherent heterogeneity of the sample. The Raman spectra of the natural and contaminated FMs showed variation in peak position and intensity, reflecting the differences in the chemical compositions of the FM samples, as illustrated in Fig. 1. The peaks corresponding to the eccrine and sebaceous components of the natural FMs, including C=O stretching from the secondary amide, CH_2_ deformation and twisting from the aliphatic chain, and C=CH deformation from squalene and unsaturated fatty acids, were clearly observed in all the spectra^31^. Additional Raman bands were observed in the spectra of the NSAID-contaminated FMs resulting from the contribution of the active tablet ingredients and other excipients present in the product. The most prominent band was attributed to ring stretching and was observed at 1598 cm^−1^ and 1629 cm^−1^ for the ketoprofen- and naproxen-containing tablets, respectively, while it was observed at 1606 cm^−1^ for the aspirin-, ibuprofen- and diclofenac-containing tablets. Table 1 shows the tentative assignment of the Raman bands of the natural FM and NSAID-contaminated FMs based on previous literature. Although Raman spectroscopy was able to detect traces of NSAIDs in the contaminated FM residues, we exploited chemometrics herein to generate a statistical model to classify and differentiate between the respective FM samples. This is the first step in identifying the components of natural FM and FMs generated after certain drug tablets were touched.

**Table 1:** Raman band assignment for the natural and NSAID-contaminated fingermarks. The asterisks (*) indicate regions selected using the GA method.

Raman band (cm^−1^)	Source	Band assignment	Ref
1655	Eccrine	C=O stretching (secondary amide)	^31^
1629*	Naproxen	Ring stretching,	^32^
1606*	Aspirin	Ring stretching and OH bending	^33^
1606*	Ibuprofen	Ring stretching	^32^
1606* and 1578	Diclofenac	Ring stretching	^37^
1598*	Ketoprofen	C–C stretching (ring)	^34^
1485*, 1420* and 1168	Naproxen	CH bending	^32^
1439	Sebaceous	CH_2_ and CH_3_ deformation (aliphatic carbon chain)	^31^
1307	Sebaceous	CH_2_ twisting (aliphatic carbon chain)	^31^
1267	Sebaceous	= CH deformation (Squalene, unsaturated fatty acid, glycerides and wax esters	^31^
1250	Diclofenac	C–C stretching CH rock,	^35^
1236	Diclofenac	C–N–C stretching, CH rock, C_7_H_2_ wagging	^35^
1207* and 1180	Ibuprofen	CH bending and OH bending,	^32^
1194*	Ketoprofen	Ring deformation and C–C stretching	^34^
1194*	Aspirin	Φ, COC stretching (Φ: ring)	^33^
1178*	Naproxen	HCC in plane bending	^36^
1159*	Aspirin	CH and OH in-plane bending	^32^
1159*	Diclofenac	CH bending (ring)	^37^
1138*	Ketoprofen	Φ -C- Φ symmetric stretch (Φ: ring)	^34^
1124 and 1078	Sebaceous	C–C stretching (aliphatic carbon chain)	^31^
1116	Ibuprofen	CH bending and OH bending,	^32^
1073* and 1045*	Diclofenac	Ring breathing	^37^
1045*	Aspirin	C–H bending	^33^
1031*	Ketoprofen	CH in-plane bending	^34^
1009*	Ibuprofen	CH in-plane bending	^32^
1002*	Ketoprofen	Ring deformation and CH_3_ rocking	^34^
1002	Eccrine	Ring breathing (phenyl alanine)	^31^
961	Naproxen	Torsion-HCCH	^36^
861*	Diclofenac	CH twisting	^37^
860*	Eccrine	Para-substituted ring vibration (Tyrosine)	^31^
748*	Naproxen	Torsion-HCCC	^36^
720*	Diclofenac	CH wagging	^35^
704*	Ketoprofen	CH out-of-plane bending	^34^
524	Naproxen	HCC in-plane bending, Torsion-HCOC, Torsion-HCCO	^36^

### Statistical analysis of Raman spectral data

**Figure 2:**
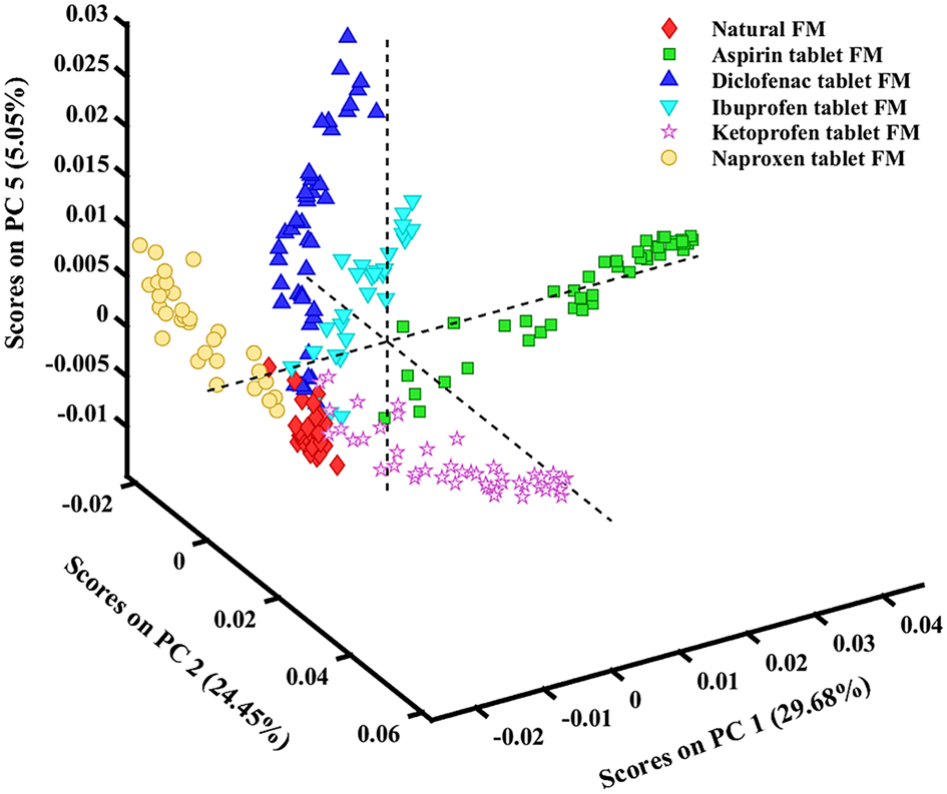
PCA scatter plot for individual Raman spectra of natural and NSAID-contaminated fingermarks.

Statistical modeling was employed to study the variation in the Raman spectra of the natural and contaminated FMs, which account for the different functional groups present in the sample, as shown in Table 1. Initially, an unsupervised learning technique, PCA model, was applied to explore the dataset and to reduce the dimensionality of the multivariate data by generating several uncorrelated variables that successfully capture the maximum variance in the dataset. Multivariate outliers were removed through PCA, defined as spectra with high Hoteling’s T^2^ and Q residuals resulting in a total of 225 spectra: 31 spectra of natural FM, 45 spectra of aspirin tablet FM, 43 spectra of diclofenac tablet FM, 28 spectra of ibuprofen tablet FM, 47 spectra of ketoprofen tablet FM and 31 spectra of naproxen tablet FM. The first five PCs described 84.5% of the total variance. For representation purposes, the PCA score plot of three PCs (PC 1, PC 2 and PC 5) for different FM samples is depicted in Fig. 2. This figure suggests that the three PCs were able to separate the dataset into six different groups corresponding to natural FM and aspirin-, diclofenac-, ibuprofen-, ketoprofen- and naproxen-contaminated FMs. However, the spectra of some of the drug-contaminated FM samples overlapped with those of the natural FM in the PCs projection. This result can be attributed to the contribution of the FM components in all the datasets. As PCA is an unsupervised statistical discrimination tool, such a contribution is expected to be prominent in the statistical model. Therefore, to provide sufficient separation between FM samples, a supervised learning technique, PLS-DA, was employed on the dataset.

### Differentiating between natural fingermarks and NSAID tablet-contaminated fingermarks using partial least squares-discriminant analysis (PLS-DA)

**Figure 3:**
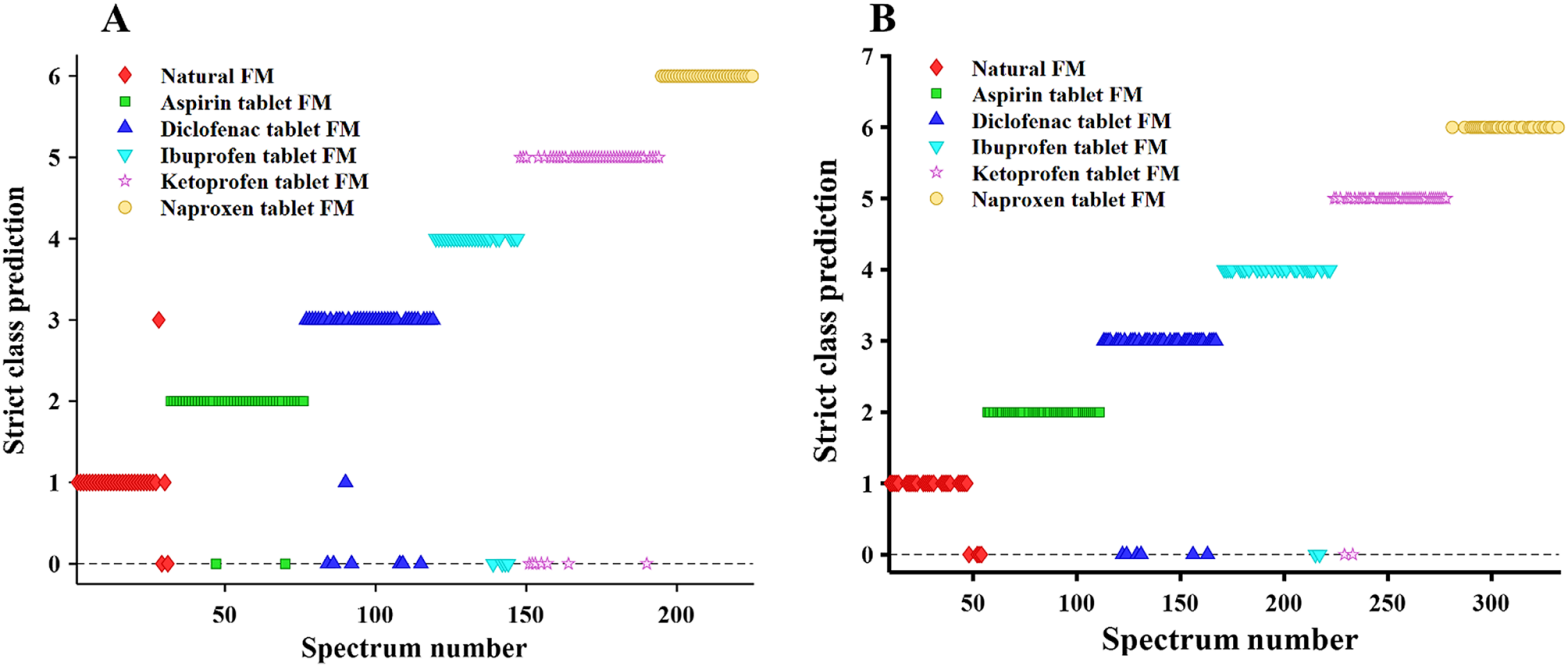
Cross validation prediction results of the PLS-DA model of each classification group using five latent variables: natural fingermark (red) and aspirin (green), diclofenac (navy blue), ibuprofen (light blue), ketoprofen (pink), and naproxen (orange) contaminated fingermarks (**A**) before applying GA and (**B**) after using the regions selected by GA.

PLS-DA is a versatile algorithm that can be employed for prediction and description as well as discrimination of variable selections. In theory, PLS-DA is a linear classification method that combines dimensionality reduction and discrimination power of the classification technique, thereby offering a high differentiation power that can be used for class prediction within the datasets^38^. Therefore, PLS-DA has shown great potential in modeling multivariate datasets for different purposes, including food analysis^39^, disease diagnosis^40^, and, more specifically, analysis of forensic evidence^41^. For instance, Lednev and coworkers pioneered the identification of all main bodily fluids for forensic purposes using PLS-DA model^42^. High levels of discrimination between human and animal blood, menstrual and peripheral blood and phenotype profiling based on bloodstain analysis have been achieved using PLS-DA model combined with ATR FTIR spectroscopy^43–45^. In the present study, we used this method to detect and identify five different drugs in contaminated FMs. We accomplished these goals by differentiating six classes of FMs, including natural FM that were free of any drugs and FMs contaminated with five different drugs. Specifically, after the spectra were baseline-corrected by automatic weighted squares, normalized by total area and mean centered, a PLS-DA model was built using five latent variables (LVs) to classify a total of 225 Raman spectra of natural and contaminated FM samples. Figure 3A shows the prediction results of cross validation (CV) based on individual spectra for different FM samples. A value of 1 corresponds to natural FM, a value of 2 corresponds to aspirin tablet-contaminated FM, a value of 3 corresponds to diclofenac tablet-contaminated FM, a value of 4 corresponds to ibuprofen tablet-contaminated FM, a value of 5 corresponds to ketoprofen tablet-contaminated FM and a value of 6 corresponds to naproxen tablet-contaminated FM, whereas a score of 0 indicated unassigned prediction. The CV prediction plot showed that 18 spectra were unassigned, and 4 spectra were misclassified, resulting in 90% correct classification of the cross validation test. Table S1 summarizes the prediction results on the PLS-DA model obtained from the CV test.

To improve the prediction results of the PLS-DA model, GA was proposed to select the regions that would be most informative for differentiating between the Raman spectra of different FM classes. GA is a machine learning technique that ultimately aims to optimize a given response function and is based on mimicking the theory of natural biological evolution. GA is a very useful method for variable selection in calibration and classification modeling, and more details about it can be found in the following article^46^. In this study, GA was carried out, and several spectral regions that noticeably contributed to the distinction between FM samples were selected, as illustrated in Table 1. Within the selected regions, the main difference can be attributed to ring stretching of the NSAID tablets at 1598–1629 cm^−1^. Regions were also selected at 1159–1207 cm^−1^ and assigned to the -CH and -OH bending of aspirin, diclofenac, ibuprofen and naproxen tablets, and at the peak at 1194 cm^−1^ was assigned to ring deformation C–C stretching of the ketoprofen tablet. Other informative bands in the 1073–1002 cm^−1^ and 704–861 cm^−1^ regions were distinctive of NSAID tablets, suggesting their significant contribution to distinguishing between the Raman spectra of different FM classes. Figure 3B demonstrates the CV prediction results of the PLS-DA model using regions selected by GA. The results were drastically improved upon applying GA, yielding 94% accuracy. Spectra of both the aspirin- and naproxen-contaminated FMs were perfectly assigned to their designated groups, while four spectra of natural FM and six spectra of diclofenac-contaminated FM were unassigned. In addition, both ketoprofen- and ibuprofen-contaminated FMs showed good separation in the model, and only two spectra were unassigned, while no misclassification was observed in the created model. This information is summarized in Table S2, which shows the confusion matrix of CV of the PLS-DA model using the strict class prediction method.

### Method validation

**Figure 4:**
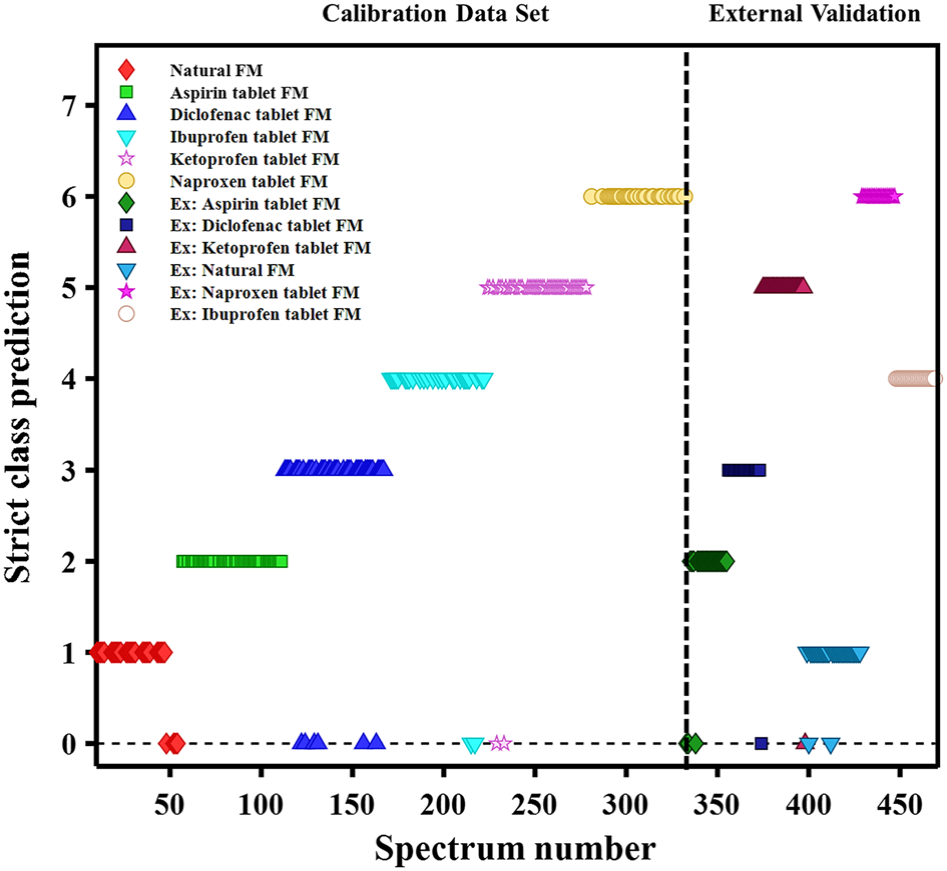
Strict prediction results of the PLS-DA classification model for natural FM- and NSAID-contaminated FMs based on five latent variables using regions selected by GA. The left side of the plot shows the calibration results based on the training dataset. The right side of the plot shows the external validation tests from an independent donor.

To further support the reliability of the created model, external validation was performed using FMs provided by a new donor. Both natural and contaminated FMs were obtained from this donor, a total of 137 spectra were loaded into the model as unknown, and each spectrum was then assigned to a specific class. Figure 4 illustrates the prediction results of the external validation, specifically, 130 spectra were correctly assigned to the respective natural and contaminated FM classes, while only seven spectra were unassigned. This means that 94% of the total external validation spectra were correctly assigned to their corresponding FM classes, indicating the excellent performance of the constructed model, as outlined in Table 2. Most importantly, over 85% of all spectra obtained for an individual FM were assigned correctly for each of the six classes of FMs. This result indicates that if we choose a threshold of 85%, then all samples used for the external validation are assigned correctly, demonstrating 100% accuracy of the developed classification model. This proof-of-concept study offers a new approach to identifying natural and drug-contaminated FM components using Raman spectroscopy and multivariate statistical analysis.

**Table 2:** A confusion matrix for individual spectra obtained for the external validation of the PLS-DA model based on five latent variables using the regions selected by the GA method.

Class predicted	Actual class					
	Natural FM	Aspirin tablet FM	Diclofenac tablet FM	Ibuprofen tablet FM	Ketoprofen tablet FM	Naproxen tablet FM
Natural FM	28	0	0	0	0	0
Aspirin tablet FM	0	20	0	0	0	0
Diclofenac tablet FM	0	0	18	0	0	0
Ibuprofen tablet FM	0	0	0	22	0	0
Ketoprofen tablet FM	0	0	0	0	23	0
Naproxen tablet FM	0	0	0	0	0	19
Unclassified	2	3	1	0	1	0

## Conclusion

This proof-of-concept study further develops Raman spectroscopy and chemometrics as a nondestructive and rapid method for the detection and identification of drugs in latent fingermarks (LFMs). Raman spectra were collected from natural FM and aspirin-, diclofenac-, ibuprofen-, ketoprofen- and naproxen-contaminated FMs obtained after the donor gently handled these tablets. Initially, PCA model was applied to identify spectral outliers, which were removed from the dataset before any further statistical analysis. Thereafter, multivariate PLS-DA and GA were employed to differentiate between natural and NSAID-contaminated FMs. The PLS-DA model was created using a training dataset and enabled an excellent separation of natural FM, aspirin-, diclofenac-, ibuprofen-, ketoprofen- and naproxen-contaminated FMs according to Venetian blind cross validation (CV). In addition, the method was externally validated using FM samples obtained from a second donor, and the results of strict class prediction showed 94% correct classification based on individual spectra. Most importantly, the individual samples showed 100% correct identification. Thus, the reported results demonstrate the great potential of Raman spectroscopy and chemometrics for the detection and identification of trace NSAIDs and potentially other drugs in LFMs. When fully developed and implemented in practical forensics, this methodology holds great promise in criminal investigations of drug overdose and handling, adulterant and suicide. Before the developed method can be applied by law enforcement agencies, further work is required to cover a wider range of drugs. In particular, we plan to expand the use of the current method and include several other drugs such as procuring and counterfeit drugs in addition to emerging synthetic cannabinoids in the future. Furthermore, the application of this method for examining other potential exogenous materials including explosives, gunshot residue, cosmetics and others in fingermarks will be also considered. Additionally, the variation in the chemical composition of the prints due to ungroomed or groomed types, potential interferences from environmental contaminants and common substrates as well as environmental conditions, including temperature, humidity, sunlight, should be addressed to simulate samples from real crime scenes.

## Supplementary Information

Supplementary Information.
