# Supplementary material for: Detection and identification of drug traces in latent fingermarks using Raman spectroscopy
Source: Sci Rep. 2022 Feb 24;12:3136. doi: 10.1038/s41598-022-07168-6 (PMC8873478; doi:10.1038/s41598-022-07168-6)
Supplement: Supplementary file 1 — Supplementary Information. [file 41598_2022_7168_MOESM1_ESM.pdf]

Supporting Material

Detection and Identification of Drug Traces in Latent Fingermarks Using Raman Spectroscopy

Mohamed O. Amin[1], Entesar Al-Hetlani[1*], Igor K. Lednev[2*]

[1] Department of Chemistry, Kuwait University, Faculty of Science, P.O. Box 5969, 13060 Safat, Kuwait

[2] Department of Chemistry, University at Albany, SUNY, 1400 Washington Avenue, Albany, NY 12222, USA

Corresponding authors: entesar.alhetlani@ku.edu.kw and ilednev@albany.edu

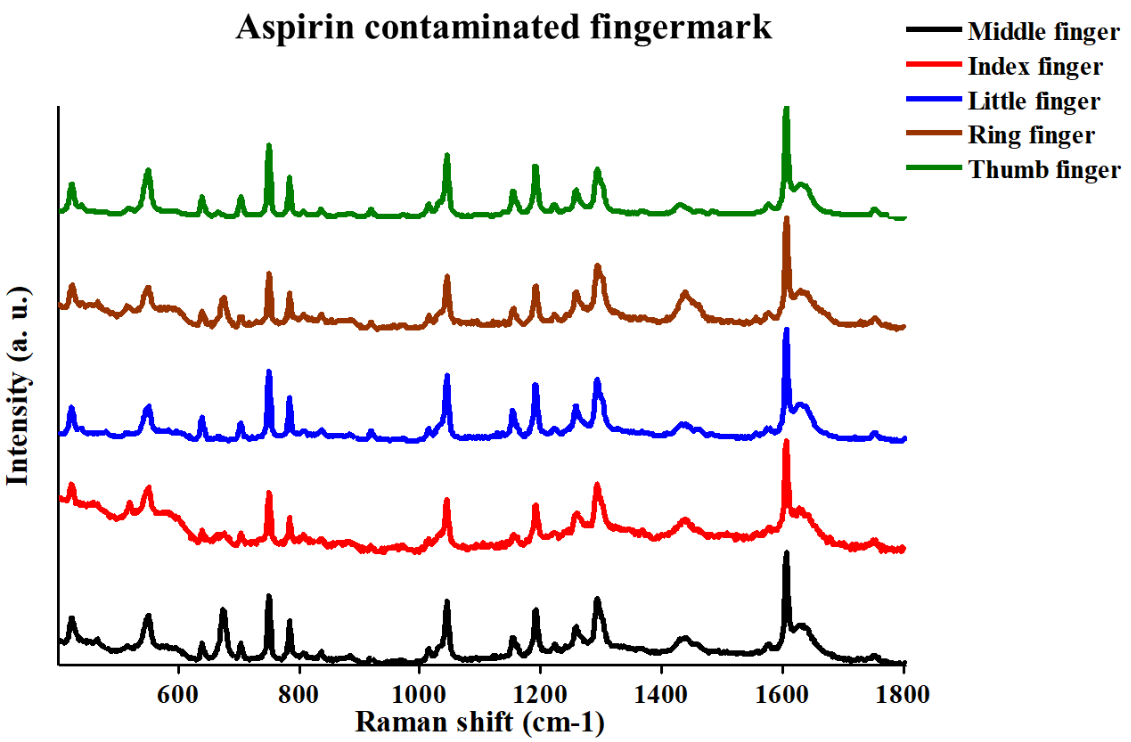

Figure S1: Average Raman spectra of aspirin contaminated fingermark obtained from five different fingers of the same donor.

Table S1: Confusion matrix of cross validation (CV) of the PLS-DA model based on five latent variable using strict class prediction method.

Class predicted	Actual class					
	Natural FM	Aspirin tablet FM	Diclofenac tablet FM	Ibuprofen tablet FM	Ketoprofen tablet FM	Naproxen tablet FM
**Natural FM**	28	0	1	0	0	0
**Aspirin tablet FM**	0	43	0	0	0	0
**Diclofenac tablet FM**	2	0	37	1	0	0
**Ibuprofen tablet FM**	0	0	0	24	0	0
**Ketoprofen tablet FM**	0	0	0	0	41	0
**Naproxen tablet FM**	0	0	0	0	0	30
**Unclassified**	1	2	5	3	6	1

Table S2: Confusion matrix of cross validation (CV) of the PLS-DA model based on five latent variable using strict class prediction method using regions selected by GA.

Class predicted	Actual class					
	Natural FM	Aspirin tablet FM	Diclofenac tablet FM	Ibuprofen tablet FM	Ketoprofen tablet FM	Naproxen tablet FM
**Natural FM**	27	0	0	0	0	0
**Aspirin tablet FM**	0	45	0	0	0	0
**Diclofenac tablet FM**	0	0	37	0	0	0
**Ibuprofen tablet FM**	0	0	0	26	0	0
**Ketoprofen tablet FM**	0	0	0	0	45	0
**Naproxen tablet FM**	0	0	0	0	0	31
**Unclassified**	4	0	6	2	2	0
